# The Utility of High‐Sensitivity Troponin to Detect Cardiomyopathy in Patients With Fabry Disease

**DOI:** 10.1002/jmd2.70008

**Published:** 2025-08-12

**Authors:** Subadra Wanninayake, Tejas Kalaria, Antonio Ochoa‐Ferraro, Ashwin Roy, Richard Steeds, Tarekegn Geberhiwot, Charlotte Dawson

**Affiliations:** ^1^ Department of Inherited Metabolic Disorders Queen Elizabeth Hospital Birmingham Birmingham UK; ^2^ Department of Chemical Pathology The Royal Wolverhampton NHS Trust Birmingham UK; ^3^ Department of Cardiology Queen Elizabeth Hospital Birmingham Birmingham UK; ^4^ Institute of Metabolism and System Research University of Birmingham Birmingham UK

**Keywords:** cardiac MRI, Fabry cardiomyopathy, high‐sensitivity troponin, late gadolinium enhancement, left ventricular hypertrophy

## Abstract

Fabry disease (FD) is an X‐linked lysosomal storage disease resulting in lysosomal accumulation of glycosphingolipids in multiple organs. In this study, we (1) compare high‐sensitivity cardiac troponins I and T (hs‐cTnI and hs‐cTnT) as markers of Fabry cardiomyopathy (FC), and (2) evaluate the role of hs‐cTn in monitoring early‐stage FC to establish if there is a threshold at which significant FC can be reliably excluded. Data were collected retrospectively from clinical records of adults with FD seen in the Inherited Metabolic Disorders service, Birmingham, UK. Patients with cardiac magnetic resonance imaging and concurrent hs‐cTnT and/or hs‐cTnI measurement(s) were included. FC was defined as the cardiac magnetic resonance imaging features of LVH and/or late gadolinium enhancement and low/pseudonormal T1. One hundred thirty‐three patients (male = 54) were included (hs‐cTn incidences = 259), 62 patients had significant FC, and 71 did not have FC. hs‐cTnI and hs‐cTnT were compared by expressing as the fractional ratio (FRTn=hs‐cTn concentration/age‐ and sex‐specific 99th percentile). The distribution of FRTnI and FRTnT was not different (median [interquartile range], 0.89 [0.52–2.02] vs. 1.02 [0.47–1.9], *p* = 0.270), including in patients with and without cardiomyopathy. In differentiating patients with FC from those without, the area under the curve (AUC) for hs‐cTn was higher (AUC = 0.974; 95% CI: 0.959–0.990; *p* < 0.001) than for FRTn (AUC = 0.897) and NT‐proBNP (AUC = 0.939). A threshold hs‐cTn < 8.5 ng/L was optimal to exclude FC (sensitivity 97.8% and negative predictive value 97.2%) with comparable performance of hs‐cTnT and hs‐cTnI at this threshold. The degree of serum hs‐cTn elevation correlated with the severity of cardiac involvement. We conclude that serial hs‐cTn monitoring is superior to NTproBNP for monitoring for early FC. cMRI scans to identify features of FC that are indicators to start treatment can be prioritised to patients with hs‐cTn > 8.5 ng/L.

**Abbreviations**
DMT: disease‐modifying treatment
ERT: enzyme replacement therapy
FC: Fabry cardiomyopathy
FD: Fabry disease
FRTn: fractional ratio of hs‐cTn (= hs‐cTn concentration/age‐ and sex‐specific 99th percentile value)
Gb3: globotriaosylceramide
GLA: alpha‐galactosidase A
LGE: late gadolinium enhancement

1

**Summary**

- A high sensitivity cardiac troponin (hs‐cTn) I and T can be used interchangeably to monitor for early Fabry cardiomyopathy. Hs‐cTn result of less than 8.5 ng/L reliably rules out cardiomyopathy.

## 2 Introduction

Fabry disease (FD) is an X‐linked inherited lysosomal storage disease (OMIM #301500) caused by pathogenic variants in the gene encoding galactosidase‐α (*GLA*, Xq22.1), leading to deficient activity of the enzyme alpha‐galactosidase A (GLA, EC 3.2.1.22) [1, 2]. The defective enzyme activity leads to the progressive accumulation of globotriaosylceramide (Gb3) in the lysosomes of affected cells, causing inflammation, apoptosis and eventually, organ dysfunction. The highly variable clinical manifestation of FD is linked to the level of residual GLA activity caused by the specific variant in the *GLA* gene [3, 4, 5, 6, 7]. In the classical phenotype, complete deficiency or severe reduction in GLA activity results in progressive multi‐organ involvement, evident from a young age. In the non‐classical phenotype, with higher residual enzyme activity, the most common manifestation is progressive cardiomyopathy.

Cardiac magnetic resonance imaging (cMRI) plays a central role in identifying early pre‐symptomatic cardiac manifestations of FD and in staging disease severity, broadly mapped to a 4‐phase model [8]. Stages 1–2 represent early cellular disruption, evident on resting ECG as an isolated finding of a short PR interval and on cardiac MRI as low signal on T1 images (‘low T1’). However, clinical focus is on timely identification of Stage 2, heralding the onset of Stage 3, where lysosomal accumulation of Gb3 causes significant inflammation and myocardial hypertrophy. This is evident on cardiac MRI as increased myocardial mass and wall thickness with preserved ejection fraction, early diastolic dysfunction and focal late gadolinium enhancement (LGE), typically in the basal inferolateral segment. The presence of one or more of these features on cardiac MRI is an indication for initiating disease‐modifying treatment (DMT), either enzyme replacement therapy (ERT) or chaperone therapy. The aim of treatment is to slow progression to Stage 4, which is characterised by advanced cardiomyopathy with fibrosis leading to heart failure and/or malignant arrhythmia and sudden cardiac death [9, 10, 11]. This stage is evident on cardiac MRI as systolic dysfunction, extensive LGE and pseudonormalised T1 with high focal or global T2 signal. In Stage 4, systolic dysfunction, LGE and malignant arrhythmia may be evident even in the absence of LVH, as the myocardium is progressively replaced with fibrotic tissue [8, 12]. Thus, monitoring of Fabry cardiomyopathy (FC) using cardiac MRI allows for early initiation of DMT to mitigate the risk of developing fibrosis with the potential to improve long‐term outcomes [13, 14]. However, in a resource‐limited healthcare system, monitoring for FC with serial cardiac MRI represents a significant cost to the system and burden for the patient. The identification of a readily available blood biomarker that could reliably exclude significant FC would allow us to prioritise cardiac MRI for patients for whom it is most likely to impact their care.

Damaged myocardial cells release cardiac troponins detectable in routine serum samples. Persistent modest elevation of high‐sensitivity cardiac troponins I and T (hs‐cTnI, hs‐cTnT) is an established biomarker for detecting subclinical myocardial damage in ischaemic and non‐ischaemic cardiomyopathies [15, 16, 17]. In FD, chronically elevated hs‐cTnI and hs‐cTnT have been described in advanced, symptomatic stages of FC [13, 18, 19]. Yet, the quantification and relative increase of each hs‐cTn type at early stages of FC and how it relates to cardiac MRI features remain outstanding areas of investigation. Furthermore, whilst hs‐cTnI and hs‐cTnT are considered equivalent for the diagnosis of acute cardiac ischemia and for evaluating cardiac remodelling after a coronary event, their equivalence in evaluating FC is not known. The aim of this study is (1) to compare hs‐cTnI and hs‐cTnT as markers of FC, and (2) to evaluate the role of hs‐cTn in monitoring early‐stage FC to establish if there is a threshold at which significant FC is likely, as an indication for further workup by cardiac MRI and for consideration of treatment initiation.

## 3 Method

### 3.1 Study Setting and Participants

This retrospective study was conducted in patients with genetically confirmed FD assessed between April 2011 and April 2024 in a multidisciplinary, multi‐professional national lysosomal storage disorders centre at University Hospitals Birmingham NHS Foundation Trust, UK. The study was conducted after formal registration with the local Clinical Audit Department as a part of a service evaluation and did not require ethics approval. Relevant demographic characteristics, clinical manifestations and results of investigations were collected retrospectively from each patient's hospital record. Patients with genetically confirmed FD and cardiac MRI with concurrent hs‐cTn (either hs‐cTnT or hs‐cTnI) measurement(s) were included in this study. FD patients for whom LGE analysis was not performed and who do not have LVH were excluded from the study to minimise false negatives arising from incomplete data.

### 3.2 Data Collection

For purposes of this study, FC was defined as cMRI features of LVH and/or LGE with either low T1 (either single region of interest or global value) or pseudonormal T1 (T1 that becomes normal in the presence of extensive LGE). Cardiac MRI was interpreted by cardiologists with expertise in reporting FD cardiac MRI as part of routine clinical practice. FD patients were classified into one of two groups according to whether cardiac MRI features of FC were present or absent. The hs‐cTn measurement was considered concurrent with cMRI if performed within 6 months prospectively or 12 months retrospectively from the time of the cMRI.

The hs‐cTnT and hs‐cTnI were measured in the institution's ISO 15189 accredited laboratory using proprietary Roche Cobas and Abbott Alinity methods, respectively. For the purposes of data analysis, hs‐cTn results below the lower reporting limit of the assay (5 ng/L for both hs‐cTnT and hs‐cTnI) were recorded as 4.9 ng/L. The hospital laboratory's hs‐cTn method transitioned from Roche Cobas hs‐cTnT to Abbott Alinity hs‐cTnI in May 2019. In paired analysis of hs‐cTnT and hs‐cTnI, the chronologically closest hs‐cTnT and hs‐cTnI results between November 2017 and October 2021 with a maximum interval of 18 months between measurements were used. To further facilitate the comparability of hs‐cTnT and hs‐cTnI results, the values were expressed as a fractional ratio of hs‐cTn (FRTn) calculated as hs‐cTn concentration/age‐ and sex‐specific 99th percentile value. The 99th percentile values for hs‐cTn were derived from a population study in the UK [20]. Data were excluded where there was perceived to be either an analytical error or interference such as macrotroponin [21, 22] causing the cardiac troponin results to be disproportionately elevated.

Serum NT‐proBNP and serum creatinine were measured by proprietary methods on Roche Cobas and Abbott Alinity analytical platforms before and after May 2019, respectively. NT‐proBNP results were compared with hs‐cTn and its derivative FRTn as biomarkers of FC. Estimated glomerular filtration rate (eGFR) was calculated from the measured creatinine initially using the MDRD equation until the laboratory changed to the chronic kidney disease‐EPI 2009 formula in November 2021. Chronic kidney disease staging was determined from the eGFR in accordance with the Kidney Disease Outcomes Quality Initiative (KDOQI) guidelines. Patients with end‐stage renal disease were excluded because this causes chronic elevation of hs‐cTn affecting the interpretation of the results [23].

### 3.3 Statistical Analysis

Statistical analyses were conducted using SPSS version 24 (IBM Corp., USA). After testing for normal distribution using the Kolmogorov–Smirnov test, continuous non‐parametric data were presented as median with interquartile range (IQR). Categorical variables were summarised in terms of frequency and percentages. To test for significant differences between two groups, categorical data were evaluated using the Chi‐square test, and non‐parametric continuous data were analysed using Mann–Whitney *U*‐tests. In paired comparisons at two time points, non‐parametric data were analysed using the Wilcoxon Signed‐Ranks Test, and categorical data were analysed using McNemar's test. The correlation between various parameters was analysed using the Spearman rank correlation (*ρ*). All tests were two‐tailed, and a *p*‐value less than 0.05 was considered statistically significant. Receiver operating characteristic (ROC) analysis was performed to assess the predictive diagnostic accuracy and determine the optimal thresholds of hs‐cTn, FRTn and NT‐proBNP for distinguishing patients with and without significant FC.

## 4 Results

### 4.1 Demographic and Clinical Characteristics of the Study Population

**FIGURE 1:**
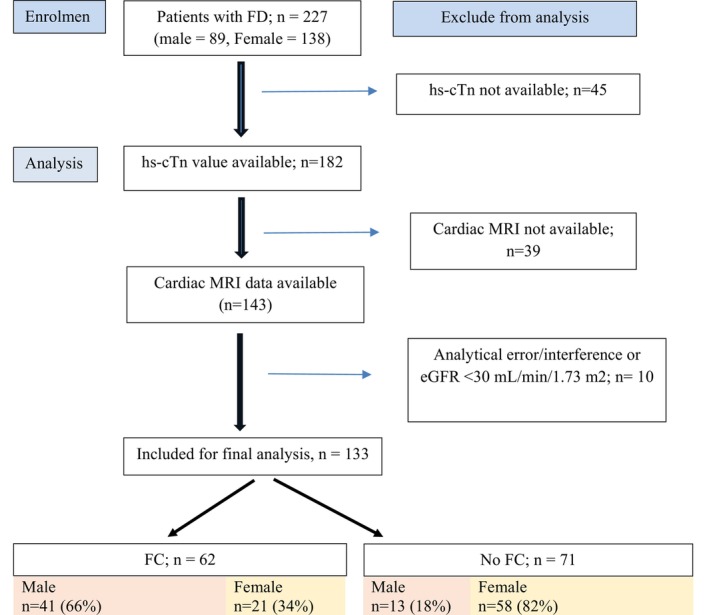
Flow diagram for the selection of patients with Fabry disease for the study. eGFR, estimated glomerular filtration rate; FC, Fabry cardiomyopathy; FD, Fabry disease; hs‐cTn, high‐sensitivity cardiac troponin; *n*, number of patients.

Of the total 227 patients with FD (89 male and 138 female), 182 patients had an hs‐cTn result available, of whom 143 had concurrent cardiac MRI. Two patients with eGFR < 30 mL/min/1.73 m^2^ were excluded, and a further eight patients were excluded because of a perceived analytical error or interference affecting the accuracy of the troponin result. The remaining 133 patients were included for final analysis (Figure 1). A total of 54 male patients, median age 55.7 (45.6–62.9) years, and 79 female patients, median age 41.9 (33.5–60.7) years, were included in the analysis, which included a total of 259 hs‐cTn measurements (173 hs‐cTnI and 86 hs‐cTnT). Of those, 62 patients (41 male, 66%) had significant FC as evidenced by the presence of LVH and/or LGE with low or pseudonormal T1 on cMRI, and 71 patients (13 male, 18%) did not have FC. FC was present in 41/54 (75.9%) of males compared with 21/79 (26.6%) females.

The demographic and clinical characteristics of patients with and without FC are summarised in Table 1. Patients with FC were older compared to patients without FC (*p* = 0.002) and the median (IQR) age of females with FC was 64.4 (60.6–71.9) compared with 57.3 (50.9–64.3) in males with FC. Serum levels of both hs‐cTn I and hs‐cTn T were higher in patients with FC compared to those without FC (median [IQR] 42 ng/L [19.5–95] vs. 5 ng/L [5–5] and 27 ng/L [15.3–39.5] vs. 5 ng/L [5–7.7] respectively; *p* < 0.001). This difference remained significant after adjusting for the age‐ and sex‐specific 99th percentile values expressed as FRTn. Median (IQR) FRTn was higher at 1.35 (0.78–2.47) in patients with FC compared to 0.53 (0.4–0.57), *p* < 0.001 in those without FC (Table 1). Lyso‐Gb3, a marker of Gb3 storage, was significantly higher in patients with cardiomyopathy than in those without (*p* < 0.001) (Table 1). NT‐proBNP was also higher in patients with FC (384 [150–1079] ng/L) compared to those without FC [54 (30.8–87) ng/L, *p* < 0.001] (Table 1).

**TABLE 1:** Demographic and clinical characteristics of patients with and without Fabry cardiomyopathy based on cardiac magnetic resonance imaging.

Parameter	Cardiomyopathy	No cardiomyopathy	*p*
	Median (IQR)/*n* = patients number	Median (IQR)/*n* = patients number	
Age (years)	60.6 (54.5–66.2)/*n* = 62	37.3 (29.3–43.4)/*n* = 71	0.002
Male age (years)	57.3 (50.9–64.3)/*n* = 41	36.7 (27.7–41.9)/*n* = 13	< 0.001
Female age (years)	64.4 (60.6–71.9)/*n* = 21	37.3 (30.7–44.7)/*n* = 58	< 0.001
Non‐classical	41/62	34/73	0.017
Laboratory data			
hs‐cTn (ng/L)	33.5 (17–78.5)	5 (5–5)	< 0.001
hs‐cTn I (ng/L)	42 (19.5–95)	5 (5–5)	< 0.001
hs‐cTn T (ng/L)	27 (15.3–39.5)	5 (5–7.7)	< 0.001
FRTn	1.35 (0.78–2.47)	0.53 (0.4–0.57)	< 0.001
FRTnI	1.4 (0.8–3.0)	0.53 (0.30–0.57)	< 0.001
FRTnT	1.15 (0.71–2.1)	0.47 (0.44–0.47)	< 0.001
NT‐proBNP (ng/L)	384 (150–1079)	54 (30.8–87)	< 0.001
eGFR (mL/min)	74.5 (61–88)	90 (87–90)	< 0.001
ACR (mg/mmol)	2.5 (1.0–8.2)	2.4 (0.8–9.3)	0.764
Lyso Gb3 (nmol/L)	5 (2.5–9.6)	1.5 (0.8–4.4)	< 0.001
Imaging findings			
LV mass index	110 (76.5–141.5)	62 (56–70)	< 0.001
LVEF	74 (66–80)	71 (66–75)	0.030
T1 value	888 (850–941)	924 (886–972)	< 0.001
Low/pseudonormal T1	62/62 (134/134 instances)	31/71 (56/125 instances)	< 0.001
Cardiovascular risk factors			
Hypertension	17	5	
Diabetes mellitus	2	4	
Dyslipidaemia	21	4	

Abbreviations: ACR, albumin to creatinine ratio; eGFR, estimated glomerular filtration rate; FRTn, fractional ratio of hs‐cTn = hs‐cTn concentration/age‐ and sex‐specific 99th percentile value; hs‐cTn I, high‐sensitivity cardiac troponin I; hs‐cTn T, high‐sensitivity cardiac troponin T; hs‐cTn, high‐sensitivity cardiac troponin; LV, left ventricular; LVEF, left ventricular ejection fraction.

### 4.2 Comparability of hs‐cTnI and hs‐cTnT in Male and Female Patients With FD

**FIGURE 2:**
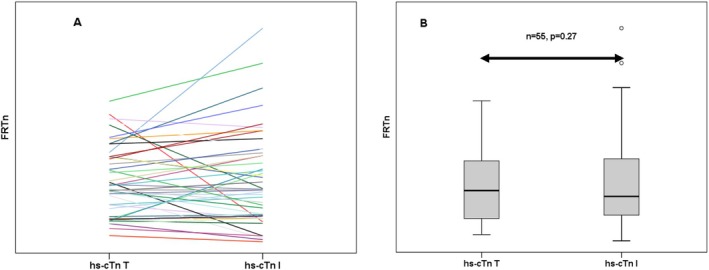
Difference between hs‐cTn I and T in patients with Fabry disease. (A) Demonstrates the fractional ratio of high‐sensitivity troponin (FRTn) in a logarithmic scale of chronologically closest high‐sensitivity cardiac troponin T (hs‐cTn T) and I (hs‐cTn I) in each individual with a maximum interval of 18 months between measurements. (B) Demonstrates FRTn in a logarithmic scale of hs‐cTn T and hs‐cTn I in patients with Fabry disease. *n*, number of patients. Box plots represent the median level with the 25th and 75th percentiles of observed.

A total of 55 patients had both hs‐cTnI and hs‐cTnT results within 18 months. Median (IQR) hs‐cTnI results (17 [5–65] ng/L) were higher than hs‐cTnT (16 [5–39] ng/L, *p* = 0.002), the distribution of FRTnI and FRTnT was not different (median [IQR], 0.89 [0.52–2.02] vs. 1.02 [0.47–1.9], *p* = 0.270) (Figure 2).

In subgroup analysis of 42 patients with both hs‐cTnI and hs‐cTnT results and a contemporaneous cMRI, there was no significant difference between FRTnT and FRTnI distributions in FD patients with cardiomyopathy (*n* = 21, *p* = 0.375) and similarly no significant difference between FRTnT and FRTnI distributions in FD patients without cardiomyopathy (*n* = 20, *p* = 0.936). Similarly, there is no difference in the distribution of FRTnT and FRTnI in male (*n* = 27, *p* = 0.74) and female (*n* = 28, *p* = 0.051) FD patients.

### 4.3 Diagnostic Accuracy of hs‐cTn for Excluding FC

**FIGURE 3:**
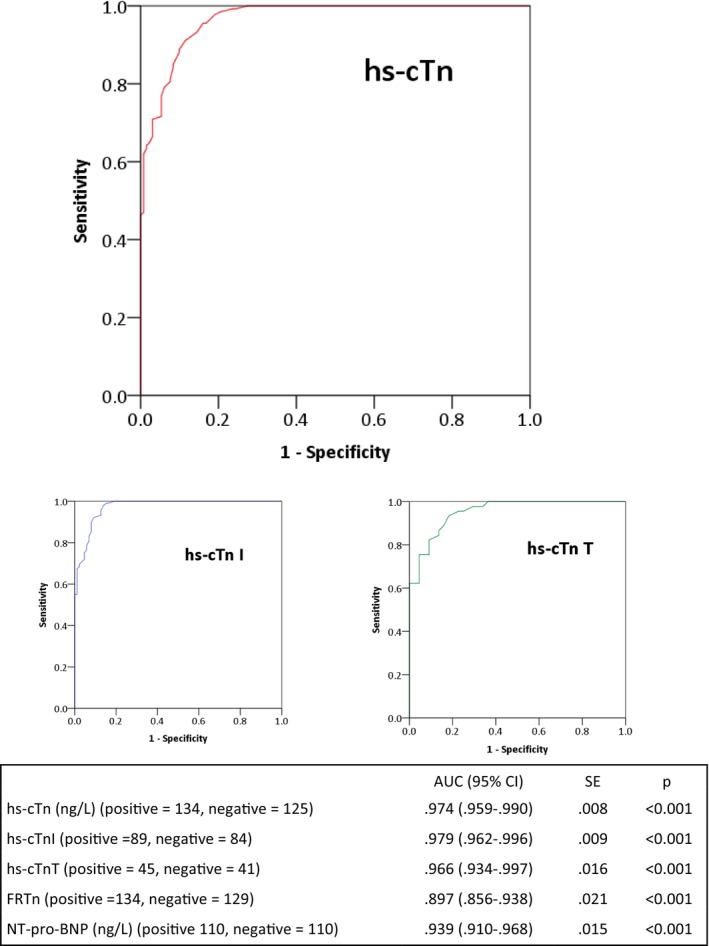
Receiver operating characteristic (ROC) curve showing diagnostic accuracy of hs‐cTn, hs‐cTnT and hs‐cTnI with cardiac MRI as a gold standard. ROC analyses of the diagnostic accuracy of high‐sensitivity troponin and NT‐proBNP for detecting Fabry cardiomyopathy using cardiac MRI as the gold standard. AUC, area under the curve; CI, confidence interval; FRTn, fractional ratio of hs‐cTn (calculated as hs‐cTn concentration/age‐ and sex‐specific 99th percentile value); hs‐cTn, high‐sensitivity cardiac troponin I; hs‐cTn, high‐sensitivity cardiac troponin; hs‐cTnT, high‐sensitivity cardiac troponin T; NT‐proBNP, B‐type natriuretic peptide; SE, standard error.

Figure 3 shows the ROC curve for hs‐cTn in differentiating patients with FC from those without. The area under the curve (AUC) for hs‐cTn was higher (AUC 0.974; 95% CI: 0.959–0.990; *p* < 0.001) than for FRTn (AUC 0.897) and NT‐proBNP (AUC 0.939).

For both troponin subtypes, hs‐cTn < 8.5 ng/L was the optimal threshold to exclude FC (sensitivity 97.8% and negative predictive value [NPV] 97.2%) with comparable sensitivity, specificity and predictive values for both hs‐cTnT and hs‐cTnI (Table 2). At this threshold, there were 3/134 instances of FC and 105/125 instances without FC, including both classical and late‐onset Fabry and in both men and women (Table 2). Conversely, hs‐cTn ≥ 8.5 ng/L detected FC with a higher positive predictive value (95%) and lower specificity (82.8%) in men compared with women (PPV = 70.6%, specificity = 84.4%).

**TABLE 2:** Performance of hs‐cTn, hs‐cTnT and hs‐cTnI at different threshold concentrations.

Parameter	Threshold level	Sensitivity (%)	Specificity (%)	PPV (%)	NPV (%)	Cardiomyopathy OR (CI)	*p*
hs‐cTn (ng/L)	8.0	98.5	83.2	86.3	98.1	326 (75–1425)	< 0.001
	8.5	97.8	84	86.8	97.2	229 (66–792)	< 0.001
	9.95	95.5	86.4	88.3	94.7	136 (52–356)	< 0.001
At threshold 8.5 ng/L							
Female		100	84.4	70.6	100		< 0.001
Male		96.9	82.8	95	88.9	152 (34–681)	< 0.001
Classical		98.1	84.1	82.8	98.3	279 (35–2238)	< 0.001
Non‐classical		97.5	83.9	89.7	95.9	203 (42–983)	< 0.001
hs‐cTnI, *n* = 173	8.0	98.9	88.1	89.9	98.7	67 (9.6–472)	< 0.001
	8.5	97.8	88.1	89.7	97.4	322 (68–1516)	< 0.001
	9.95	95.5	89.3	90.4	94.9	177 (52–598)	< 0.001
At threshold 8.5 ng/L							
Female	*n* = 94	100	86.6	75	100		< 0.001
Male	*n* = 79	96.8	94.1	98.4	88.9	480 (40–5635)	< 0.001
Classical	*n* = 80	97.1	84.4	82.9	97.4	184 (21–1577)	< 0.001
Non‐classical	*n* = 93	98.1	92.3	94.6	97.3	636 (63.6–6358)	< 0.001
hs‐cTn T, *n* = 86	8.0	97.8	73.2	80	96.8	120 (17.7–979)	< 0.001
	8.5	97.8	75.6	81.5	96.9	136 (17–1121)	< 0.001
	9.95	95.6	80.5	84.3	94.3	88.7 (17.6–445)	< 0.001
At threshold 8.5 ng/L							
Female	*n* = 38	100	79.3	60	100		< 0.001
Male	*n* = 48	97.2	66.7	89.7	88.9	70 (6.8–713)	< 0.001
Classical	*n* = 43	100	83.3	82.6	100		< 0.001
Non‐classical	*n* = 43	96.2	64.7	80.6	91.7	45.8 (4.9–427)	< 0.001

Abbreviations: hs‐cTn I, high‐sensitivity cardiac troponin I; hs‐cTn T, high‐sensitivity cardiac troponin T; hs‐cTn, high‐sensitivity cardiac troponin; *n*, number of instances; NPV, negative predictive value; OR (CI), odd ratio (confidence interval); PPV, positive predictive value.

In subgroup analysis, hs‐cTn increased as FC progressed, with results higher in the group with cardiac MRI evidence of both LGE and LVH with low/pseudonormal T1, compared with the group with either basal inferolateral LGE with low T1 and without LVH or early localised LVH with low T1, but without LGE (median [IQR], 36 [22.2–84.3] ng/L, *n* = 46 vs. 18 [11–40] ng/L, *n* = 12, *p* = 0.002 or 24 [9.8–40.5], *n* = 4, *p* = 0.14) (Figure S1). In the group with basal inferolateral LGE with low T1, but no LVH, 9/12 were female, and all patients with localised LVH with low T1, but no LGE were male.

### 4.4 Correlation of Serum hs‐cTn Levels With Other Parameters

In Spearman's correlation coefficients, hs‐cTn levels correlated moderately with LV mass index (CC = 0.72, *p* < 0.001) and weakly with lyso‐Gb3 (CC = 0.41, *p* < 0.001) and LVEF (CC = 0.13, *p* = 0.35). In addition, NT‐proBNP moderately correlated with LV mass index (CC = 0.62, *p* < 0.001) and weakly with LVEF (CC = 0.14, *p* = 0.45).

## 5 Discussion

Cardiac involvement is one of the hallmarks of clinically significant FD. In routine clinical practice, all patients have a cMRI and concurrent hs‐cTn and NT‐proBNP measurement at diagnosis. Thereafter, hs‐cTn and NT‐proBNP are measured at every appointment, and cMRI is repeated every 3–5 years if FC is not present, or more often if it is present. cMRI features of early FC are embedded in UK national guidance as an indication to initiate DMT and are also well understood for monitoring established FC. Additionally, it has been implicitly understood that an intra‐individual increase in hs‐cTn and NT‐proBNP on serial measurements over time is an adverse prognostic indicator. However, there was no established biomarker threshold at which FC could be considered significant, and to date, hs‐cTn and NT‐proBNP measurement has primarily played a supportive role to cMRI for monitoring established FC.

Timely introduction of DMT slows progression of FC. This means that identifying FC at an early‐stage is a priority for the management of asymptomatic patients with FD. However, performing regular cMRIs to detect FC is resource‐intensive, and for some patients, it is an unpleasant experience or not tolerated. Furthermore, because cMRI features of FC are the primary indication for initiating treatment, if significant FC develops in the 3–5‐year period between cMRI scans, treatment may be delayed by several years. Hence, in this study, we aimed to establish whether there is an hs‐cTn or NT‐proBNP threshold at which FC could be reliably excluded. In clinical practice, the results could then be used to guide the optimal timing of the next cMRI with the potential benefit that patients for whom treatment is indicated are identified earlier, whilst low‐risk patients are not subjected to unnecessarily frequent scans.

The study was conducted on a cohort of 133 patients with FD, and the main findings were as follows: (1) hs‐cTn was superior to NT‐proBNP for distinguishing patients with FC from those without; (2) the optimal hs‐cTn threshold is < 8.5 ng/L, ruling out FC with sensitivity 97.8% and NPV 97.2%; and (3) at this threshold, there is no difference in performance between hs‐cTnI and hs‐cTnT. Together, these findings indicate that serum hs‐cTn is a promising biomarker for detecting early FC.

Previous studies have demonstrated that both elevated troponin I and T correspond with the presence of myocardial injury in FD [13, 18]. However, the location of molecules and the amounts of bound and free forms of troponin T and I in the myocardial cells differ [24] and hence, the amount and timing of their release after cardiac injury could differ depending on the aetiology, severity and acuteness of the cardiac insult. To the best of our knowledge, our study is the first to measure both subtypes in the same patient with FD. This was fortuitously only available to us because our laboratory transitioned from Roche Cobas hs‐cTnT to Abbott Alinity hs‐cTnI during the study period. However, because this study was conducted using data collected as part of routine clinical care, this meant that for each patient, there was an interval of up to 18 months between hs‐cTnT and hs‐cTnI measurements which we acknowledge as a limitation to the study potentially affecting the comparability of the two biomarkers. Nevertheless, importantly, at the proposed hs‐cTn threshold of 8.5 ng/L, the sensitivity, specificity and negative predictive values of both troponin subtypes were comparable suggesting that at this threshold, hs‐cTnT and hs‐cTnI can be used interchangeably to reliably rule out significant FC. A further prospective study would ideally compare hs‐cTnT and hs‐cTnI measured simultaneously to increase our confidence that the two assays can be used interchangeably.

A previous study demonstrated that hs‐cTnI at a higher threshold of 40 ng/L identified LVH of non‐Fabry aetiology and Fabry‐related LVH [13]. This is the first study demonstrating the utility of cardiac troponin at a lower threshold to rule out early FC. Our findings suggest that serial hs‐cTn measurement can be used in place of serial cMRI as a monitoring tool for patients who, at the time of initial diagnosis of FD, do not have cMRI evidence of FC or who have low T1 only. On this basis, we propose that cMRI could be reserved for patients with hs‐cTn ≥ 8.5 ng/L. Prioritising patients for cMRI whose hs‐cTn is ≥ 8.5 ng/L could reduce the numbers of cMRI scans performed on low‐risk patients, reducing the burden of care for patients less likely to benefit whilst freeing up the resource for patients for whom a timely cMRI could potentially alter their disease course by identifying features of FC that would prompt earlier initiation of DMT. If this proposed threshold level of hs‐cTn could apply to filter higher‐risk patients for cMRI in our cohort, 40.5% (105/259 instances) of cMRI would be avoided.

In a subgroup of patients with moderate FC as evidenced by low T1 and either LGE or LVH (but not both) there was a discrepancy between men and women. The majority (9/12) of patients with low T1 and inferolateral LGE without LVH were women, whilst all patients (4/4) with low T1 and LVH but without LGE were men. These sex differences in cMRI findings were comparable to published data [8] and highlight the importance of using either LVH or LGE (or both) as evidence of FC and indicators to start treatment. It also highlights the essential role of cMRI and the limitations of using echocardiography alone as the imaging modality to identify FC. Our data have shown that hs‐cTn reliably distinguishes patients with no cMRI features of FC or low T1 only from those with either LGE or LVH (AUC 97.4) and is superior to NT‐proBNP (AUC 93.9).

The current study did not have the statistical power to identify the hs‐cTn concentration associated with myocardial storage and dysfunction before the development of cMRI evidence of LVH. However, the hs‐cTn value did show a moderate significant correlation with LV mass quantified on cMRI. Additionally, the degree of hs‐cTn elevation increased in line with cMRI features, with the lowest results in patients with no cMRI features of FC or low T1 only, intermediate values in the cohort with either localised LGE or localised LVH, and the highest values recorded in the group who had LVH and LGE. This is in keeping with previous studies that showed the degree of hs‐cTnT elevation correlated with the development of fibrosis as FC progresses through Stage 3 to Stage 4 and correlates positively with the extent of LGE [18, 19, 25].

Whilst the mechanisms of troponin release in FC remain speculative, it is widely accepted that it arises from inflammation caused by lysosomal Gb3 storage leading to myocardial injury, apoptosis and ultimately fibrosis, which disrupts the electrical properties of cardiomyocytes [26]. In our study, the degree of elevation of lyso‐Gb3 was higher in patients with FC compared to the group without cardiac involvement and positively correlated with hs‐cTn, supporting the concept that Gb3 storage is the underlying driver of troponinaemia and the development of FC. Hence, any detection of hs‐cTn above the threshold of 8.5 ng/L, which is lower than has been conventionally considered a significant indicator of myocardial injury, is an indicator of FC and may provide insight into the extent of cardiac lysosomal Gb3 storage.

The retrospective study design has certain limitations in addition to those already mentioned; in particular, the potential for bias because the datasets may be incomplete or not representative of all patients across the disease spectrum. For example, patients at the extremes of their disease course may be excluded for being either too well or too sick to attend clinic or scan appointments. Although this study was done in a single centre and included a large cohort of patients with FD, including significant numbers with and without cardiomyopathy, a further prospective multi‐centre study is warranted. Nevertheless, we suggest that it does allow us to draw meaningful conclusions about the real‐world utility of hs‐cTnI and hs‐cTnT for monitoring this rare disease.

## 6 Conclusion

In patients with FD, serum hs‐cTn < 8.5 ng/L reliably rules out significant FC. Cardiac MRI features of FC may be present in patients with hs‐cTn as low as 8.5 ng/L, which is significantly lower than the 40 ng/L value conventionally considered an indicator of significant myocardial injury. At this threshold, both serum hs‐cTnT and hs‐cTnI had similar performance in the evaluation of cardiac manifestations of FD and can be used interchangeably depending on local test availability. Additionally, the magnitude of hs‐cTn elevation correlates loosely with the severity of cardiac involvement. Taken together, serial hs‐cTn assessment appears to be a reasonable approach for detecting early myocardial involvement in patients with genetically confirmed FD.

## Author Contributions

Subadra Wanninayake, Tejas Kalaria, Tarekegn Geberhiwot and Charlotte Dawson conceived the study and drafted the manuscript. Subadra Wanninayake and Antonio Ochoa‐Ferraro were involved in data collection. Ashwin Roy and Richard Steeds were involved in reporting cardiac MRI. Subadra Wanninayake and Tejas Kalaria analysed the data. All authors made contributions to writing and reviewing the final manuscript.

## Consent

The authors have nothing to report.

## Conflicts of Interest

Subadra Wanninayake, Tejas Kalaria, Antonio Ochoa‐Ferraro, Ashwin Roy and Charlotte Dawson declare they have no conflicts of interest. Tarekegn Geberhiwot and Richard Steeds have received sponsorship to attend meetings and conferences from drug companies, Amicus, Sanofi and Takeda, and unrestricted research grants from drug companies, Amicus, Sanofi and Takeda.

## Supporting information


Figure S1.

## Data Availability

Data are available on reasonable request.
