# Supplementary material for: The Utility of High‐Sensitivity Troponin to Detect Cardiomyopathy in Patients With Fabry Disease
Source: JIMD Rep. 2025 Aug 12;66(5):e70008. doi: 10.1002/jmd2.70008 (PMC12343053; doi:10.1002/jmd2.70008)
Supplement: Supplementary file 1 — Figure S1. [file JMD2-66-e70008-s001.docx]

**Supplementary figure 1: Distribution of hs-cTn related to cardiac MRI findings in the study population**

**
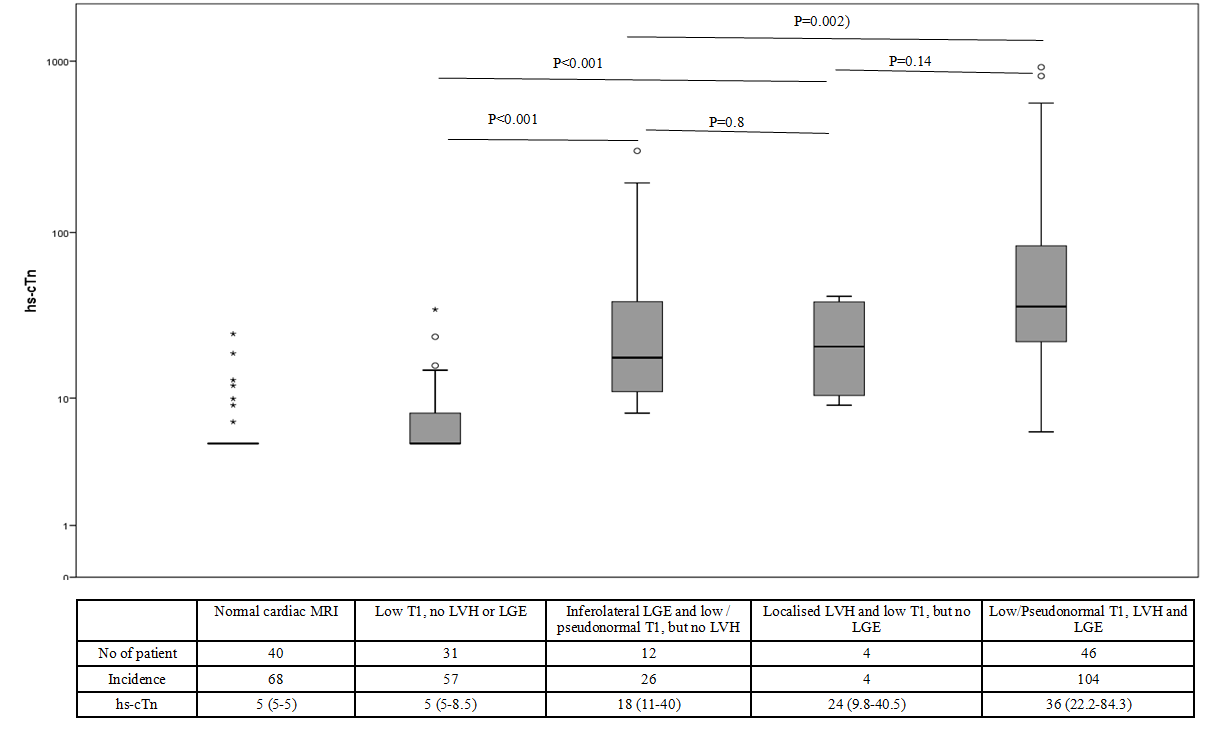
**

Figure demonstrates hs-cTn level in logarithmic scale in patients with Fabry disease in different severity level of cardiomyopathy: Box plots represent median level with 25th and 75th percentiles of observed data. hs-cTn; high sensitive cardiac troponin, LVH; left ventricular hypertrophy, LGE; late gadolinium enhancement.
